# Qualitative study on shared decision making in cystitis management in general practice

**DOI:** 10.3399/BJGPO.2023.0179

**Published:** 2024-07-24

**Authors:** Tessa MZXK van Horrik, Annelies Colliers, Marco H Blanker, Eefje GPM de Bont, Antoinette A van Driel, Bart J Laan, Suzanne E Geerlings, Roderick P Venekamp, Sibyl Anthierens, Tamara N Platteel

**Affiliations:** 1 Department of Internal Medicine-Infectious Diseases, Amsterdam UMC, University of Amsterdam, Amsterdam Institute for Infection and Immunity, Amsterdam Public Health, Amsterdam, The Netherlands; 2 Department of Family Medicine & Population Health, University of Antwerp, Antwerp, Belgium; 3 Department of Primary and Long-term Care, University Medical Center Groningen, University of Groningen, Groningen, The Netherlands; 4 CAPHRI, Department of Family Medicine, Maastricht University, Maastricht, The Netherlands; 5 Erasmus University Medical Center Rotterdam, Rotterdam, The Netherlands; 6 Department of General Practice & Nursing Science, Julius Center for Health Sciences and Primary Care, University Medical Center Utrecht, Utrecht University, Utrecht, The Netherlands

**Keywords:** qualitative research, cystitis, general practice, decision making, shared, urinary tract infections, primary health care, primary care nursing

## Abstract

**Background:**

Cystitis is commonly treated with antibiotics, although non-antibiotic options could be considered for healthy non-pregnant women. Shared decision making (SDM) can be used in cystitis management to discuss the various treatment options but is not frequently applied in general practice.

**Aim:**

To identify barriers and facilitators for applying SDM in cystitis management in general practice.

**Design & setting:**

Qualitative explorative research in general practice with healthcare professionals (HCPs; GPs and GP assistants) and healthy non-pregnant women with a recent history of cystitis (patients).

**Method:**

Individual semi-structured interviews were conducted between May and October 2022. We applied a combination of thematic and framework analysis.

**Results:**

Ten GPs, seven GP assistants, and 15 patients were interviewed. We identified the following three main barriers and one key facilitator: (1) applying SDM is deemed inefficient; (2) HCPs assume that patients expect antibiotic treatment and some HCPs consider non-antibiotic treatment inferior; (3) patients are largely unaware of the various non-antibiotic treatment options for cystitis; and (4) HCPs recognise some benefits of applying SDM in cystitis management, including reduced antibiotic use and improved patient empowerment, and patients appreciate involvement in treatment decisions, but preferences for SDM vary.

**Conclusion:**

SDM is infrequently applied in cystitis treatment in general practice owing to the current focus on efficient cystitis management that omits patient contact, HCPs’ perceptions, and patient unawareness. Nevertheless, both HCPs and patients recognise the long-term benefits of applying SDM in cystitis management. Our findings facilitate the development of tailored interventions to increase the application of SDM, which should be co-created with HCPs and patients, and fit into the current efficient cystitis management.

## How this fits in

Although cystitis symptoms can resolve spontaneously, this condition is among the most common reasons for antibiotic prescribing in general practice, which in turn induces the emergence of antimicrobial resistance (AMR). Previous research showed that healthy women with cystitis are willing to postpone antibiotic treatment provided they are well-informed about alternative options; for example, through shared decision making (SDM). We found that healthcare professionals (HCPs) infrequently apply SDM in cystitis treatment in general practice, because of (among other reasons) a need for efficient cystitis management, HCPs’ assumptions about patients’ preferences, and HCPs’ views on non-antibiotic treatment options. Nevertheless, both women with cystitis and HCPs recognise the potential benefits of applying SDM in cystitis treatment, including less antibiotic use and improved patient empowerment.

## Introduction

Urinary tract infections (UTIs) are a prime reason for GP visits and antibiotic prescribing.^1–3^ A recently published meta-analysis comparing strategies to reduce antibiotics with immediate antibiotic treatment for uncomplicated UTIs, including cystitis, showed that immediate antibiotic treatment decreased the symptom burden on day two compared with non-antibiotic treatment.^4^ Besides, immediate antibiotic treatment slightly reduced the risk of pyelonephritis (absolute risk difference 1.6%) compared with placebo or non-steroidal anti-inflammatory drugs (NSAIDs).^4,5^ However, antibiotic use contributes to the emergence of AMR.^6^

Avoiding antibiotic use through self-care advice or a delayed prescription strategy reduces not only the risk of AMR but also of antibiotic-related side effects, especially since symptoms tend to settle spontaneously in 50% of healthy women with cystitis.^7–11^ The Dutch general practice guideline for UTIs thus recommends that GPs discuss the following three management options with these patients: a wait-and-see policy (with analgesics if needed); a delayed antibiotic prescription; or an immediate prescription.^5^ The options are considered equivalent and should be discussed with the patient.

SDM allows HCPs and patients to make decisions together by using the best available evidence.^12^ Previous studies show that SDM is an effective approach to match management options to patients’ needs and preferences, and thereby reduce antibiotic prescriptions in general practice.^13,14^ Both GPs and women are open to non-antibiotic treatment options for cystitis,^15–17^ and acknowledge the added value of patients being involved in the treatment decision-making process.^18^

Despite the potential benefits, SDM is not routinely adopted in everyday practice for healthy non-pregnant women with cystitis, as recently highlighted by the ‘Appropriate Care’ project, initiated by the Dutch National Health Care Institute.^19^ To tailor interventions aimed at promoting SDM for the treatment of cystitis in healthy non-pregnant women presenting to general practice, we conducted an in-depth exploration with HCPs and patients of the barriers and facilitators for implementing SDM.

## Method

### Study design and setting

We conducted an explorative qualitative study in general practice in The Netherlands using semi-structured remote interviews with GPs, GP assistants, and women with a recent history of cystitis. In this study, we defined cystitis as UTI without systemic symptoms, such as fever, malaise, chills, costovertebral pain, or signs of sepsis or delirium.^5^ We used the Consolidated Criteria for Reporting Qualitative Research (COREQ) checklist for reporting this study.^20^

In The Netherlands, the GP assistant usually uses triage to identify systemic symptoms and risk factors for a complicated disease course (for example, diabetes mellitus, immunocompromised state, urinary tract anomalies, or pregnancy) and performs urinalysis for diagnostic purposes. For women without risk factors for a complicated disease course (healthy non-pregnant women) and in the absence of systemic symptoms, the GP assistant typically discusses their findings with the GP or immediately initiates antibiotic treatment that is later approved by the GP.^2,18^

### Participant selection and recruitment

Eligible HCPs (GPs and GP assistants) were recruited through the professional network of the project group, and social media groups for GPs (LinkedIn, Facebook, and Telegram Messenger). We used purposive sampling to achieve heterogeneity in age, work experience, type of general practice, and working region. Healthy non-pregnant women with a recent history of cystitis (that is, <2 years) were recruited through the participating GPs and social media. Those interested were contacted by telephone or email and received a study information leaflet. We explored age, the region of residency, UTI history, and education level. All participants received a financial reimbursement for their interviews.

### Data collection and analysis

After obtaining verbal informed consent from participants, the primary researcher (TvH) conducted individual semi-structured interviews by video (Microsoft Teams) or telephone. We used separate semi-structured topic guides for HCPs and patients (Supplementary files S1 and S2). The topic guides were developed following the Consolidated Framework For Implementation Research^21^ based on sensitising concepts from literature review and expert input from the multidisciplinary research team. The topic guides were pilot tested with one GP, one GP assistant, and two patients. During the interviews, participants’ experiences with current management and the application of SDM in cystitis treatment, and factors contributing to or hindering this application were explored (see Supplementary file S1 and Supplementary file S2 for further details).

The interviewer (TvH), a physician trained in qualitative interviewing, had no professional or hierarchical relationship with the participants. Ten GPs, seven GP assistants, and 15 patients were interviewed. All interviews were video- and/or audio-recorded and verbatim transcribed and field notes were made.

We applied a combination of thematic and framework analysis,^22,23^ supported by NVivo (version 12.7.0 for Mac). Two researchers (TvH and AC) independently coded the first three interview transcripts of both HCPs and patients, line-by-line, after which they discussed the initial codes and identified potential categories and subcategories. The interview guides were refined throughout the iterative process of data collection and analysis. Subsequent coding was done by the primary researcher (TvH). To enhance the quality of the data analysis, researcher triangulation was carried out, including a discussion of the data analysis at several stages among the wider research team (Supplementary file S3). Involving researchers with a variety of clinical and research experiences in UTIs and/or other infectious diseases across various healthcare settings ensured broad reflexivity on the results (Supplementary file S3). We had regular discussions within the research team (TvH, AC, and TP), and constantly compared the understandings and interpretations with the original data.

We continued data collection until we reached data sufficiency.^24,25^ Data triangulation was enhanced by including both HCPs and patients.

## Results

Thirty-two interviews were conducted between May and October 2022. Participants’ characteristics are summarised in Tables 1 2. The mean duration of the interviews was 50 minutes for the HCPs (range 30–60 minutes), and 30 minutes for patients (range 18–46 minutes). All but one patient (P1) received antibiotics for a cystitis episode.

**Table 1.** Characteristics of participating healthcare professionals (GPs and GP assistants)

ID	Age category, years		Sex	Work experience, years	Region of work place		Type of general practice^a^	
***GPs***								
**GP1**	26–35		F	0–5	South		Duo	
**GP2**	36–45		M	11–15	South		Group	
**GP3**	36–45		F	6–10	Middle		Duo	
**GP4**	46–55		M	11–15	Middle		Healthcare centre	
**GP5**	56–65		F	>25	North		Duo	
**GP6**	26–35		F	0–5	South		Solo	
**GP7**	46–55		M	11–15	Middle		Healthcare centre	
**GP8**	46–55		M	>25	North		Group	
**GP9**	36–45		F	6–10	Middle		Solo	
**GP10**	36–45		F	6–10	South		Solo	
***GPAs***								
**GPA1**	56–65		F	11–15	Middle		Solo	
**GPA2**	18–25		F	0–5	Middle		Group	
**GPA3**	26–35		F	11–15	North		Duo	
**GPA4**	36–45		F	16–20	South		Group	
**GPA5**	18–25		F	0–5	Middle		Various	
**GPA6**	26–35		F	11–15	Middle		Group	
**GPA7**	46–55		F	0–5	Middle		Healthcare centre	

^a^In The Netherlands, general practices can be run by one GP (solo), or by multiple GPs (duo or group). In healthcare centres various healthcare professionals (for example, a GP, dietician, a physical therapist, and/or a psychologist) work within the same building. F = female. GPA = GP assistant. M = male

**Table 2.** Characteristics of participating healthy non-pregnant women (patients) with a recent history of cystitis

ID	Age category, years	Region of residency	Education	UTI history; frequency in lifetime^a^
**P1**	56–65	North	Bachelor degree	1–2
**P2**	18–25	Middle	Secondary education	1–2
**P3**	26–35	South	Secondary vocational	Recurrent
**P4**	66–75	North	Secondary vocational	≥3
**P5**	56–65	South	Secondary vocational	1–2
**P6**	46–55	Middle	Master degree	1–2
**P7**	36–45	Middle	Master degree	≥3
**P8**	26–35	Middle	Master degree	≥3
**P9**	46–55	South	Bachelor degree	Recurrent
**P10**	56–65	South	Bachelor degree	Recurrent
**P11**	56–65	South	Secondary education	≥3
**P12**	46–55	South	Secondary vocational	≥3
**P13**	56–65	North	Secondary vocational	≥3
**P14**	66–75	North	Secondary vocational	≥3
**P15**	18–25	South	Secondary education	≥3

^a^Recurrent UTIs = ≥3 UTIs in the past year (from the time of participation). Patients designated ‘≥3 in lifetime’ = patients who experienced three or more UTIs in their lifetime, but not within 1 year of participation. These, therefore, they did not meet the criteria for ‘recurrent’ UTI. P = patient.

### Themes

**Figure 1.**
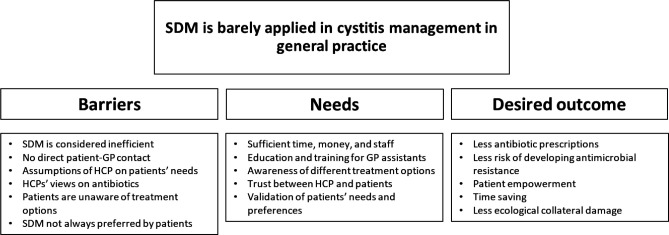
Identified barriers and facilitators for shared decision making in cystitis management. HCP = healthcare professional. SDM = shared decision making. UTIs = urinary tract infections

We identified the following four main themes: (1) current cystitis management focuses on efficiency by omitting direct patient–GP contact; (2) HCPs assume their patients’ needs and expectations; (3) patients are unaware of the opportunities in cystitis treatment; and (4) Some proposed benefits and solutions to SDM barriers in (non-antibiotic) cystitis treatment. The barriers and facilitators for applying SDM in cystitis treatment in general practice are listed as sub-themes and categorised in Figure 1.

#### Current cystitis management focuses on efficiency by omitting direct patient–GP contact

Most HCPs are satisfied with the current UTI management and its organisation, mainly owing to its efficiency (GP4). In cases of cystitis, where SDM could be used, patients typically communicated with the GP assistant, who is actually not trained in using SDM. Recommendations for diagnostics and treatment are mostly documented in practice guidelines or local protocols, in which non-antibiotic treatment options for healthy non-pregnant women are not always included. Further, most otherwise healthy non-pregnant women are usually not actively involved in the treatment decision-making process in everyday practice (P14 in theme 3).

Owing to time constraints and practical convenience, remote consultations are the preferable communication method for both HCPs and patients. This is further enhanced owing to a lack of privacy on the front desk when consulting a GP assistant face to face (GP10).

Therefore, some HCPs believed applying SDM would be too time-consuming in an already time-pressured work environment. Some GPs felt that SDM should be a process in which the different treatment options for cystitis are discussed with the patient in terms of benefits, harms, and risks of a complicated disease course. They also expressed that patients should be provided with sufficient time to carefully weigh the pros and cons of the various options, and this may require multiple telephone calls. Other GPs considered SDM as a process in which the patient is informed about the different types of antibiotic treatment rather than discussing all available management options including non-antibiotic treatment:

*‘In fact, the system is designed to confirm or rule out a UTI as quickly as possible. The wait-and-see policy is indeed rarely used* (…) *If you consider how many urines are delivered daily, it can be between six and ten, yeah, so, we just have to go through the process as quickly as possible, because there are so many other time-consuming tasks to attend to* (…) *so you have to set it up in a way that takes least time.* (…) *And, honestly, it all seems simple* (…) *but if you want to do it properly, it takes time, and well, you make choices.’* (GP4)

*‘We have a front desk and a waiting room that are not properly separated. So, discussing thoroughly* [when the GP assistant assesses a patient’s history and urine sample] *is sometimes inconvenient in relation to privacy.’* (GP10)

#### Healthcare professionals assume their patients’ needs and expectations

Most HCPs indicated that patients expect antibiotic treatment once cystitis is confirmed (GP3). However, most HCPs do not validate these assumptions. Other HCPs indicated that they make an assessment of the patient’s burden, based on reported symptoms, and propose antibiotic treatment or a wait-and-see policy without actively involving the patient in the treatment decision-making process (GPA4).

Most HCPs do not recognise the added value of a delayed antibiotic prescription, because patients can simply contact the general practice during office hours if a wait-and-see policy fails. Further, some HCPs preferred a wait-and-see policy over a delayed antibiotic prescription to curb AMR (GP6). Nevertheless, HCPs believed delayed prescriptions are convenient before weekends and holidays to prevent women from calling out-of-hours primary care should symptoms not settle or worsen over time. Other HCPs considered non-antibiotic treatment options, including analgesics, inferior to antibiotics, which discouraged them from using SDM (GP6 and GP8). Most HCPs regarded general recommendations, including ample fluid intake and proper bladder voiding, as standard recommendations in addition to antibiotic treatment:

*‘When someone calls and delivers a urine sample, and usually their symptoms have existed for a few days already, I assume, yeah, you’re not doing this without good reason. So, the suggestion of antibiotic treatment is always convenient. Well, not just convenient, also logical.’* (GP3)

*‘It* [proposing a non-antibiotic treatment option] *kind of depends on someone’s symptoms. It* [wait-and-see policy] *is not often proposed as standard treatment. It also depends on the GP I consult (…) That* [a wait-and-see policy in combination with analgesics] *is not proposed to everyone and is not always proposed by the GPs either.’* (GPA4)

*‘I think you want to keep control* [over antibiotic use] *or something like that; so you know whether people have used it* [delayed prescription] *and how the symptoms proceed. And yes, during the weekend or when someone goes on holiday, you don’t want them to go to the emergency GP centre. And you want to prevent someone from having to go consult a doctor abroad. So, I am more likely to use it* [delayed prescription] *in those situations. But yes, during office hours, I think: "Oh well, they can just call" and if they still want antibiotics, they will do that.’* (GP6)

*‘Look, you need a proper alternative (…) If, for example, if a patient comes by because she has symptoms, yeah, and wants something for that, yeah, an antibiotic* [treatment] *is available in case of a true infection. And then I think, yeah, I don’t know if that* [offering alternative non-antibiotic treatment options] *is going to be of added value.*’ (GP8)

#### Patients are unaware of the opportunities in cystitis treatment

Most patients expressed that they were not actively involved in the treatment decision-making process when they had cystitis, but emphasised that they would have liked to be. Further, patients indicated their preference for SDM varied, depending on previous experiences with UTIs and the burden of cystitis on their daily life activities (P2 and P1). In addition, women who had recurrent UTIs and were familiar with the wait-and-see policy and general advice, often demanded antibiotics when contacting the general practice, because they had already tried non-antibiotic options before seeking medical help. Nevertheless, most patients indicated that their needs and expectations were not explored when contacting the general practice for their suspected cystitis.

Some patients favoured non-pharmacological options to resolve cystitis symptoms and some indicated antibiotics can harm their bodies. However, none had received information about the potential harms or side effects of antibiotics, nor about alternative treatment options (P14). Owing to a lack of knowledge and previous experiences, patients often assumed antibiotic treatment was required (P3). Patients further indicated that, if deciding on non-antibiotic treatment initially, a delayed prescription would give them more confidence in being able to continue their daily life activities (P9):

*‘I think we just need to consider the options and see how we can get rid of it* [cystitis] *as quickly as possible if it really bothers me. But other than that, I don’t think there’s much else to discuss with the doctor.*’ (P2)

*‘And the last time I was at the GP’s, it was more that I was still in a process and wanted advice on that. So yeah, sometimes I just need someone to think along.’* (P1)

*‘Yes, that really was an eye-opener, for the first time in years in fact, that someone said "Have you tried acetaminophen?" (…) And therefore, because alternative options were never discussed, I genuinely believed that antibiotics were the only cure for this* [UTIs]*.’* (P3)

Participant: *‘Look, they immediately say "come on by and deliver a urine sample" (…) Well, then you deliver it at the general practice and you have to call them after 4 o’clock for the results (…).’* Interviewer: *‘And when you deliver the urine sample, do they discuss anything with you?’* Participant: *‘No (…) they only tell you to call back after 4 o’clock in the afternoon for the test results.’* (P14)

*‘At that time, I could collect it* [delayed prescription]*. Eventually, I didn’t have to use the prescription, I could drink it [cystitis] away and it resolved (…) but I went* [on holiday] *with a lot more confidence because I just had that regimen in my bag and thought, yeah, if it persists, I can use that cure.*’ (P9)

#### Some proposed benefits and solutions to SDM barriers in (non-antibiotic) cystitis management

Most GPs indicated they used SDM in the management of recurrent cystitis and complicated UTIs, but not for incident cystitis, partly because GPs tend to play a bigger role in the management of recurrent UTIs than in incident cases. Some GPs would support more frequent application of SDM. Their motives included reducing antibiotic prescriptions and improving patient education, and some had an overall positive attitude towards SDM (GP10, and GP6 theme 2). Further, some HCPs indicated that empowering patients with information through SDM might save time in the long term, because of a reduction of future contacts with the general practice for cystitis.

Some HCPs noticed patients appreciated being involved in the treatment decision-making process, and patients support these observations (GPA5, P12). Additionally, some patients desired additional information about the likelihood of developing complications, particularly pyelonephritis, as well as preventive measures to reduce the risk of developing UTIs (P6).

Most GPs and GP assistants indicated that the GP assistants could play an important role in applying SDM in cystitis treatment, provided they acquire the necessary training to apply SDM, which most GP assistants are willing to receive (GP2). The mutual trust between the GP and GP assistant ensured that the GP assistant could take on this task (GP2). Besides, patients trusted GP assistants to discuss the clinical assessment with the GP, knowing they could always request an appointment with their GP if needed (P1 and P12). Most HCPs believed they could probably adjust or rearrange some of the current steps in cystitis management to enable the use of SDM, while simultaneously maintaining efficiency in general practice; for example, providing information about treatment options when the patient delivered a urine sample instead of during the phone consultation when both the result and treatment plan are discussed:

*‘I think less antibiotic use, that would be the benefit. And of course, the patient’s self-sufficiency (…) when women see that it* [cystitis] *can resolve spontaneously and they are empowered to handle it themselves, that, of course, saves a bit of time and busyness with us.’* (GP10)

*‘I notice they* [patients] *appreciate it when you can offer several* [treatment] *options. It comforts them and it also ensures they call less often. They choose and try one option first and after that, they think: I’m going to try the other option she* [the GP assistant] *suggested. In this way, people become more self-sufficient.’* (GPA5)

*‘Actually, I was never offered an alternative. So, I don’t know if there are really alternatives instead of a cure* [antibiotics] *(…) I think it’s important to know: ‘What are the disadvantages, the advantages, and what are the risks?’ And I can certainly discuss that with her* [my GP]*.*’ (P12)

*‘As a patient, you’d kind of want to hear: "What are the chances of me feeling well again in a reasonable amount of time without antibiotics?" And what is the risk of A: having a longer symptom duration, or B: that it will become a complicated UTI and things will take a turn for the worse?*’ (P6)

*‘Well, at least having a conversation about "So, what do you already know about it* [cystitis]*, have you ever had it before, how did the previous episode go", yeah, the assistant can surely ask simple things like that. And knowing that a wait-and-see policy is possible, that is something they* [GP assistant and patient] *could decide together.’* (GP2)

*‘And I received a call from the assistant once, and she said "I just discussed with the GP during the break and in fact, we want to do this and that" (…) so that, you see, they work with short lines of communication and they’re also supervised carefully (…) the GP monitors the answers of the assistant (…) that gives me a good feeling.’* (P1)

*‘If you have a question or you have complaints like that* [UTI-related]*, then it’s very easy to, very nice if you can just talk to someone on the phone because then you can ask some more in-depth questions or you can give someone more context (…) the moment you are really worried, I think I would make an appointment with the GP.’* (P12)

## Discussion

### Summary

In The Netherlands, HCPs experienced several barriers to using SDM, such as time restrictions, their assumptions about patient expectations, and their views on non-antibiotic treatment. Most HCPs are not eager to adjust their work process to implement SDM, because they consider SDM time-consuming. However, they acknowledged some benefits of SDM such as less antibiotic use and time-saving in the long term. In general, GP assistants appeared more willing to apply SDM in cystitis treatment than GPs. Importantly, healthy non-pregnant women with a recent history of cystitis are mostly unaware of non-antibiotic treatment options for cystitis, but expressed a preference for being involved in the treatment decision-making process.

### Strengths and limitations

A main strength of our study is the triangulation of perspectives from GPs, GP assistants, and patients. We gained in-depth information about barriers and facilitators for the application of SDM in cystitis management in general practice. Our study has some limitations. Our study population of patients predominantly consisted of women with a history of multiple UTIs. Patients with a history of multiple UTIs tend to have different needs and expectations than those experiencing a first episode.^26–28^ However, we also included some patients with a history of few UTIs, and the variations in experience with UTIs provided a broad perspective of the results, leading to data sufficiency for patients with few UTIs as well. Further, since all of the women interviewed identified as White European, results may not be fully applicable to those from a different background, who may have different attitudes towards SDM. Additionally, although purposive sampling through professional networks and participating GPs could lead to some limitations, we achieved data sufficiency in our results concerning our research aim. Therefore, future research could include focus groups to explore the current findings from a group perspective. Further, online qualitative interviewing, increased by the COVID-19 pandemic, offers flexibility, cost savings, and participant comfort. Of course, depending on the topic and the sensitivity of the questions asked, we need to reflect whether contextual data are essential to the study and the importance of physical proximity should be evaluated. For this study, we considered reflection on the contextual data and body language unnecessary. Last, the transferability of the results of qualitative research depends on the context. Since the healthcare system in The Netherlands is similar to that in countries such as the UK, the findings of this study are applicable to similar primary care systems. Further, we provided detailed information about the methodology to help others decide whether the results are transferable to their context.

### Comparison with existing literature

The management of cystitis in general practice is designed to be efficient and since HCPs considered applying SDM to be time-consuming, they see limited opportunities for applying SDM in cystitis treatment. Interestingly, the results of a meta-analysis showed that SDM does not necessarily prolong the duration of medical consultations.^29^ We identified barriers to applying SDM in cystitis management that are similar to those of previous studies investigating effective antibiotic prescribing and the use of SDM in cystitis management in general practice.^18,30,31^ Additionally, we identified facilitators for applying SDM in cystitis treatment, such as training GP assistants to apply SDM, HCPs’ perceptions that the application of SDM could be time-saving in the long-term, and patients’ varying preferences for the use of SDM. Further, in line with a previous study showing that GPs act on patient pressure, preferences and symptoms, HCPs in our study believed they met patients’ expectations, although they usually did not validate their assumptions.^32^ Moreover, some patients in our study indicated their lack of awareness of alternative treatment options for cystitis led to the perception that antibiotics were the only cure for cystitis.^18,28^

These findings suggest a vicious cycle in which HCPs presume that women always want antibiotics and as a result do not discuss non-antibiotic treatment options with them, leading to women assuming that cystitis always requires antibiotic treatment. As a result, they often expect to receive antibiotics. Patients’ expectations in UTI management in general practice often arise during their first experience and affect subsequent episodes,^28,33^ suggesting that discussing the various treatment options during their first contact with cystitis is important.

### Implications for research and practice

In accordance with other studies, we found that some patients indicated they would not prefer pharmacological treatment if they had known that cystitis could resolve spontaneously, assuming that antibiotics and analgesics could harm your body.^16,28,34^ These factors would have been discussed if SDM was used. Applying SDM in cystitis treatment in general practice has the potential to substantially reduce antibiotic use for this condition, as patients indicated that they are willing to avoid antibiotic treatment if they are involved in the treatment decision-making process.^14,17,28^ Thus, applying SDM in cystitis treatment is not only beneficial with regard to AMR, but also may improve patient engagement and autonomy, and influence future patterns of help-seeking behaviour.^34^

The recommendation to apply SDM in cystitis treatment in healthy non-pregnant women was incorporated in the updated Dutch general practice guideline for UTIs in 2020. However, in the summary of this guideline, non-antibiotic treatment options are listed under ‘counselling’ instead of ‘treatment’.^5^ Modification of the guideline at this point may facilitate the implementation of SDM.

In contrast to the management of most other acute infections in The Netherlands, where patients are typically referred to the GP after triage, cystitis management involves GP assistants running most tasks. The GP assistant is not trained to apply SDM, which might contribute to the infrequent use of SDM for the treatment of cystitis. The current study shows that HCPs trust that GP assistants could play a key role in the implementation of SDM in cystitis management, after having received sufficient training. Further, many patients struggle with the burden of UTIs in their daily lives,^35,36^ and are keen to learn more about UTIs and the treatment options, empowering them to influence treatment decisions through SDM. Thus, future interventions should be collaboratively developed with both HCPs and patients to fully empower all stakeholders: for example, through training GP assistants to apply SDM; logistical changes, such as dedicated time and space; or educating both HCPs and patients about non-antibiotic treatment options for cystitis. For these interventions, it is essential that they fit into the current efficient UTI management in general practices.

In conclusion, SDM is infrequently applied in cystitis management in general practice. This is partly because of the focus on efficient cystitis management, HCPs considering non-antibiotic treatment inferior, and HCPs’ presumptions about patients’ preferences. Simultaneously, women with a recent history of cystitis are often unaware of the different treatment options for cystitis, and their need for SDM can vary based on their UTI experiences and the impact on their daily life activities. Nevertheless, both HCPs and patients recognise the benefits of applying SDM. Therefore, tailored interventions that improve the implementation of SDM in UTI management in general practice are required.

## Data

The unidentified dataset relied on in this article is available from the corresponding author on reasonable request.
